# m6A RNA methylation regulator-related signatures exhibit good prognosis prediction ability for head and neck squamous cell carcinoma

**DOI:** 10.1038/s41598-022-20873-6

**Published:** 2022-09-29

**Authors:** Yujia Zhai, Lian Zheng

**Affiliations:** 1 Department of Oral Medicine, The First Affiliated Hospital of Zhengzhou University, Zhengzhou, 450052 Henan People’s Republic of China; 2 Department of Oral and Maxillofacial Surgery, The First Affiliated Hospital of Zhengzhou University, Zhengzhou, 450052 Henan People’s Republic of China

**Keywords:** Cancer, Biomarkers

## Abstract

Head and neck squamous cell carcinoma (HNSCC) has become the sixth most common malignant disease worldwide and is associated with high mortality, with an overall 5-year survival rate of less than 50%. Recent studies have demonstrated that aberrantly expressed m6A regulators are involved in multiple biological and pathological processes, including cancers, but the specific mechanisms of m6A regulators in HNSCC are not well elucidated. In this study, we adopted The Cancer Genome Atlas (TCGA)-HNSCC database and performed a consensus clustering analysis to classify the HNSCC samples. Least absolute shrinkage and selection operator (LASSO) regression was applied to construct an m6A signature-based HNSCC risk prediction model. Cell type identification based on estimating relative subsets of RNA transcripts (CIBERSORT) algorithms was adopted to evaluate the immune cell infiltration level in the tumor microenvironment. Based on the expression of m6A regulators in HNSCC, we identified two clusters, cluster 1 (C1) and cluster 2 (C2). C2 showed a better prognosis than C1 and was mainly enriched in the HIPPO, MYC, NOTCH, and NRF signaling pathways. We constructed an m6A signature-based risk score model and classified patients into high- and low-risk score subgroups. The high-risk-score group showed poor clinical characteristics, higher immune infiltration levels, higher chemokine and chemokine receptor expression levels, and lower immune checkpoint gene expression than the low-risk-score subgroup. In conclusion, our comprehensive analysis suggests that the m6A signature-based risk score might function as a good prognostic predictor. Our study may provide novel therapeutic clues and help predict the prognosis of HNSCC.

## Introduction

Methylation of adenosines at the N6 position (m6A) has been shown to be the most prevalent modification of eukaryotic mRNAs^1^. Dynamic and reversible m6A modification is coordinated through installation by methyltransferases (“writers”), eradication by m6A eraser demethylases (“eraser”), and recognition by binding proteins (“readers”). m6A is the most abundant type of mRNA nucleotide modification and plays diverse roles in physiological development and pathophysiological processes, including cancers.

m6A writers include METTL3, METTL14, WTAP, and KIAA1429, and m6A erasers include FTO and ALKBH5. m6A readers, which are proteins that recognize *m6A* and regulate mRNA processing, include YTHDC1, YTHDC2, YTHDF1, YTHDF2, YTHDF3, HNRNPA2B1, IGF2BP1, IGF2BP2, and IGF2BP3. In tumor biology, m6A modification plays crucial roles in tumorigenesis, proliferation, and metastasis^2^. Reporters have revealed that m6A RNA methylation mediates non-small cell lung cancer proliferation and progression^3^. Liu et al. demonstrated that m6A has oncogenic functions and promotes gastric cancer progression^4^.

Aberrantly expressed m6A regulators also function as potential biomarkers in cancers^5–7^. Studies have shown that KIAA1429 upregulation predicts a poor prognosis in colorectal cancer^6^. Through bioinformatics analysis, Guo et al. revealed that decreased m6A regulator expression is related to greater immune cell infiltration and better survival in pancreatic cancer^5^. The evidence also indicates that METTL14 downregulation in renal cell carcinoma patients is associated with malignant characteristics and predicts a poor prognosis^7^. These studies suggest that m6A regulators might modulate the tumor microenvironment and exhibit prognostic prediction efficacy in HNSCC.

Increasing evidence demonstrates that m6A in the tumor immune microenvironment plays a critical role in the pathogenesis of various cancers. m6A modification also functions in homeostasis and tumor immunosurveillance by promoting the antitumor activity of NK cells in the tumor microenvironment^8^. Low m6A regulator-based scores are associated with a better prognosis in small-cell lung cancer after adjuvant chemotherapy^9^. In addition, this low-score cohort exhibits more CD8+ T-cell infiltration and is more responsive to cancer immunotherapy^9^. Nevertheless, the expression pattern and pathophysiological role of m6A in HNSCC remain largely unknown.

In this study, we performed bioinformatics analysis, classified HNSCC patients into subtypes with different scores and comprehensively explored the m6A signature-associated clinical features, mutations, phenotype-related gene clusters, and immune infiltration. We also constructed and validated an m6A regulator-based risk score model. Our study may provide novel clues for HNSCC prognosis and immunotherapy predictions.

## Results

### Copy number variation (CNV) and whole-exome single-nucleotide polymorphism (SNV) analyses of m6A regulators in HNSCC

Genomic alterations have a great impact on tumor development, progression, and therapeutic responses. Aberrant m6A regulator alterations are closely related to malignant tumor activities. To investigate the alterations in m6A regulators, we explored SNVs and CNVs in HNSCC from TCGA. The results revealed alterations in m6A regulator CNVs, as shown in Table 1, and RBM15 accounted for 1.01% amplification, and 47.92% of deletion rate, the landscape of regulators was listed. The proportion of YTHDF3 amplification was highest, reaching 20.88%. The various m6A regulator CNVs are illustrated in Supplementary Fig. 1. We also identified the SNVs of m6A regulators and recognized alterations in 86 (16.93%) of the 508 total samples, being the more prevalent alterations in KIAA1429 and LRPPRC (Supplementary Fig. 2).

**Table 1:** m6A modification proportions among 20 genes.

Classification	Regulators	Duplication	Deletion	CNV_sum	Amplification %	Deletion%
Writer	METTL3	958	56	1094	7.31	5.12
	METTL14	991	87	1092	1.28	7.97
	RBM15	989	92	1092	1.01	47.92
	RBM15B	766	325	1092	0.09	29.76
	WTAP	1003	62	1092	2.47	5.68
	CBLL1	974	25	1092	8.52	2.29
	ZC3H13	908	162	1092	2.01	14.84
Eraser	ALKBH5	983	78	1093	2.93	7.14
	FTO	999	72	1117	4.12	6.45
Readers	YTHDC1	994	64	1095	3.38	5.84
	YTHDC2	929	151	1096	1.46	13.78
	YTHDF1	959	20	1092	10.35	1.83
	YTHDF2	1023	55	1093	1.37	5.03
	YTHDF3	851	13	1092	20.88	1.19
	IGF2BP1	1044	11	1092	3.39	1.01
	HNRNPA2B1	947	18	1092	11.63	1.65
	HNRNPC	963	56	1100	7.36	5.09
	FMR1	1013	5	1093	6.86	0.46
	LRPPRC	1034	14	1094	4.2	1.28
	ELAVL1	990	79	1095	2.37	7.21

### m6A regulators are differentially expressed and associated with prognosis

**Figure 1:**
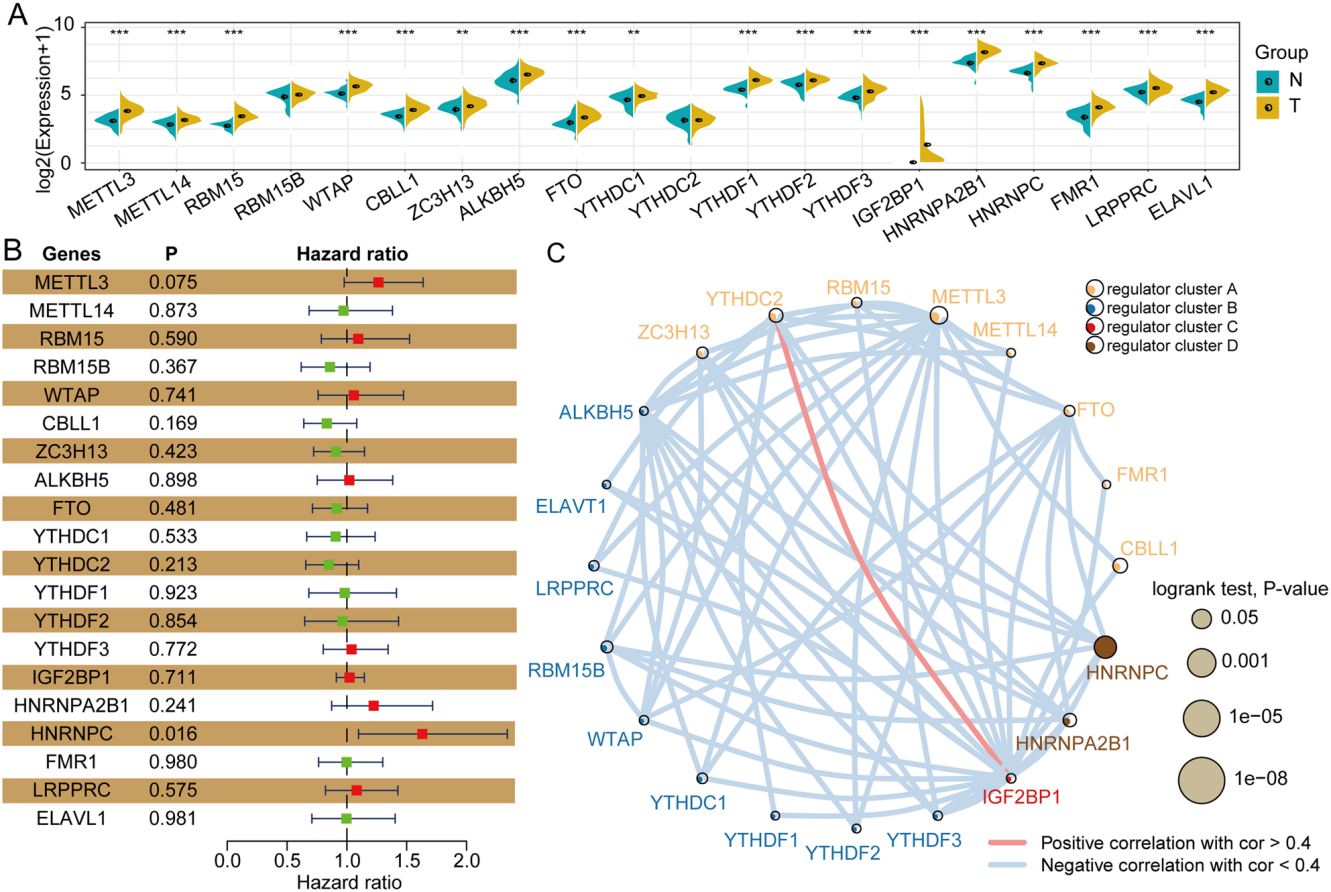
Most m6A regulators are significantly differentially expressed in HNSCC tumor tissues and are associated with patient prognosis. (**A**) m6A regulators were significantly increased in tumor tissues compared with normal tissues. (**B**) Univariate Cox analysis forest map of regulators. (**C**) Construction of the m6A-associated regulatory network. The data were analyzed by t tests: *p < 0.05; **p < 0.01; ***p < 0.001.

m6A modifications have a close relationship with tumor progression. To investigate the m6A regulator expression levels, we adopted the genomic profiles of 20 selected m6A regulators. The results revealed that 18 of these 20 genes showed significant differences between tumor tissues and paired normal tissues (Fig. 1A). To investigate whether the m6A regulator status is associated with clinical outcomes, we adopted univariate Cox regression models for the clinical follow-up data. The results revealed that HNRNPC exhibits a close relationship with the overall survival time of the patients and might play key roles in tumor progression (p = 0.016) (Fig. 1B). To better investigate the connection between m6A regulators and prognosis, we classified these 20 genes using the Euclidean method with the “dist” function based on the expression levels of these 20 genes. We then classified these 20 m6A regulator genes into four regulator clusters, namely, regulator cluster A, regulator cluster B, regulator cluster C, and regulator cluster D, based on their genomic expression levels. These results suggest that clusters with similar biological functions might present negative correlations. The red line represents positive correlations between YTHDC2 and IGF2BP1, and other clusters connected with gray lines are negatively correlated each other (Fig. 1C).

### m6A signature-based subtype

**Figure 2:**
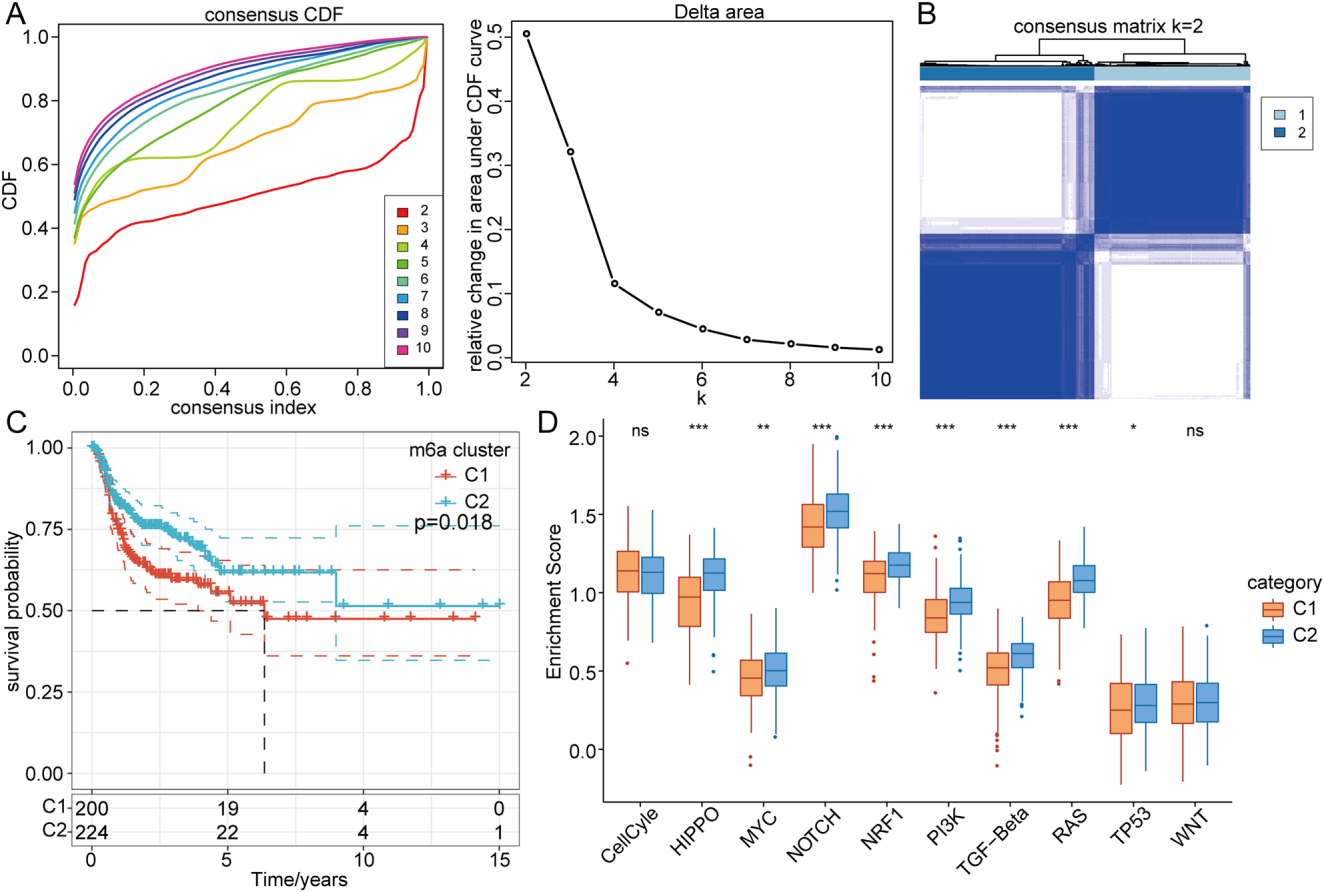
Construction of m6A-related subtypes. (**A**) CDF curve and CDF curve delta area of the TCGA HNSCC cohort. (**B**) T Sample clustering heatmap, consensus K = 2. (**C**) KM curve of the two subtypes in the TCGA-HNSCC cohort. (**D**) Estimation of the scores of different subtypes in 10 abnormal tumor pathways. (**E**) Proportions of immune cells in different subtypes.

Distinct m6A regulator-based clusters were characterized by markedly different prognoses. To better understand the m6A signatures, samples were clustered based on RNA-seq data through unsupervised clustering using ConsensusClusterPlus. We used the cumulative distribution function (CDF) and found that the clustering result obtained with k = 2 was relatively stable (Fig. 2A,B). The results demonstrated that cluster 1 exhibited a poorer prognosis than C2 (p = 0.018, Fig. 2C). We also constructed disease-specific survival (DSS), disease-free interval (DFI) and progression-free interval (PFI) curves to evaluate the prognosis of HNSCC patients. The C2 cluster showed significantly improved DSS (p = 0.032; Supplementary Fig. 3A) and PFI (p = 0.014, Supplementary Fig. 3B). The DFI (p = 0.67) showed no significant difference between the C1 and C2 clusters (Supplementary Fig. 3C). Our evaluation of the differences in tumor-associated pathway scores between the two subtypes revealed that the C2 cluster was significantly enriched in the HIPPO, MYC, NOTCH, NRF1, TGF-beta, PIK3, RAS and TP53 pathways (Fig. 2D).

### Clusters C1 and C2 show significantly different tumor mutation burdens (TMBs) and alterations

**Figure 3:**
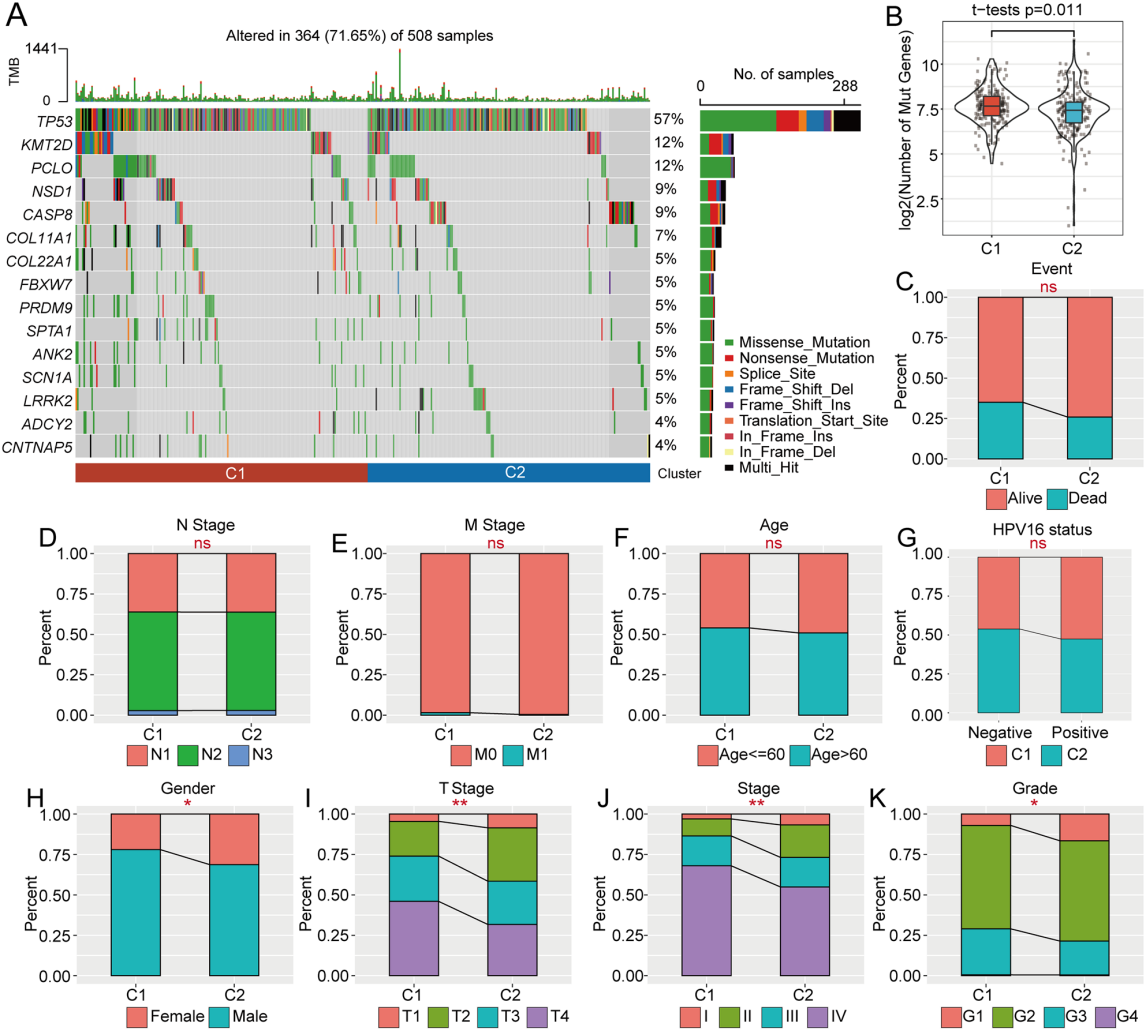
m6A modification subtypes show a close relationship with common genomic mutations. (**A**) Significantly altered genes and signatures in the C1 and C2 subgroups. (**B**) The TMB differed between the C1 and C2 subgroups. (**C**) Number of mutated genes between the C1 and C2 subgroups. (**D**–**K**) The m6A-associated subgroups C1 and C2 presented significantly different clinical feature distributions.

Different m6A regulator-based clusters were also characterized by significantly different TMBs and alterations. To characterize the TMBs in the two clusters, we analyzed the distribution pattern of the TMBs. The TMBs showed that the alterations occupied 71.65% of all samples (364 of 508) (Fig. 3A). The results indicated that C1 showed a higher TMB than C2 (p = 0.011, Fig. 3B).

### Clinical characteristics of the C1 and C2 clusters

Distinct m6A regulator-based clusters also featured significantly different clinical characteristics. To evaluate the clinical feature distribution in the two subtypes, we found no significant difference in the survival status, M stage, N stage, age, or HPV16 status (Fig. 3C–G), but significant differences in sex, T stage, stage, and tumor grade were detected (Fig. 3H–K). These results suggest that m6A signatures are closely related to clinical characteristics. The detailed clinical information is illustrated in Supplementary Table 1.

### Identification of m6A-associated DEGs

**Figure 4:**
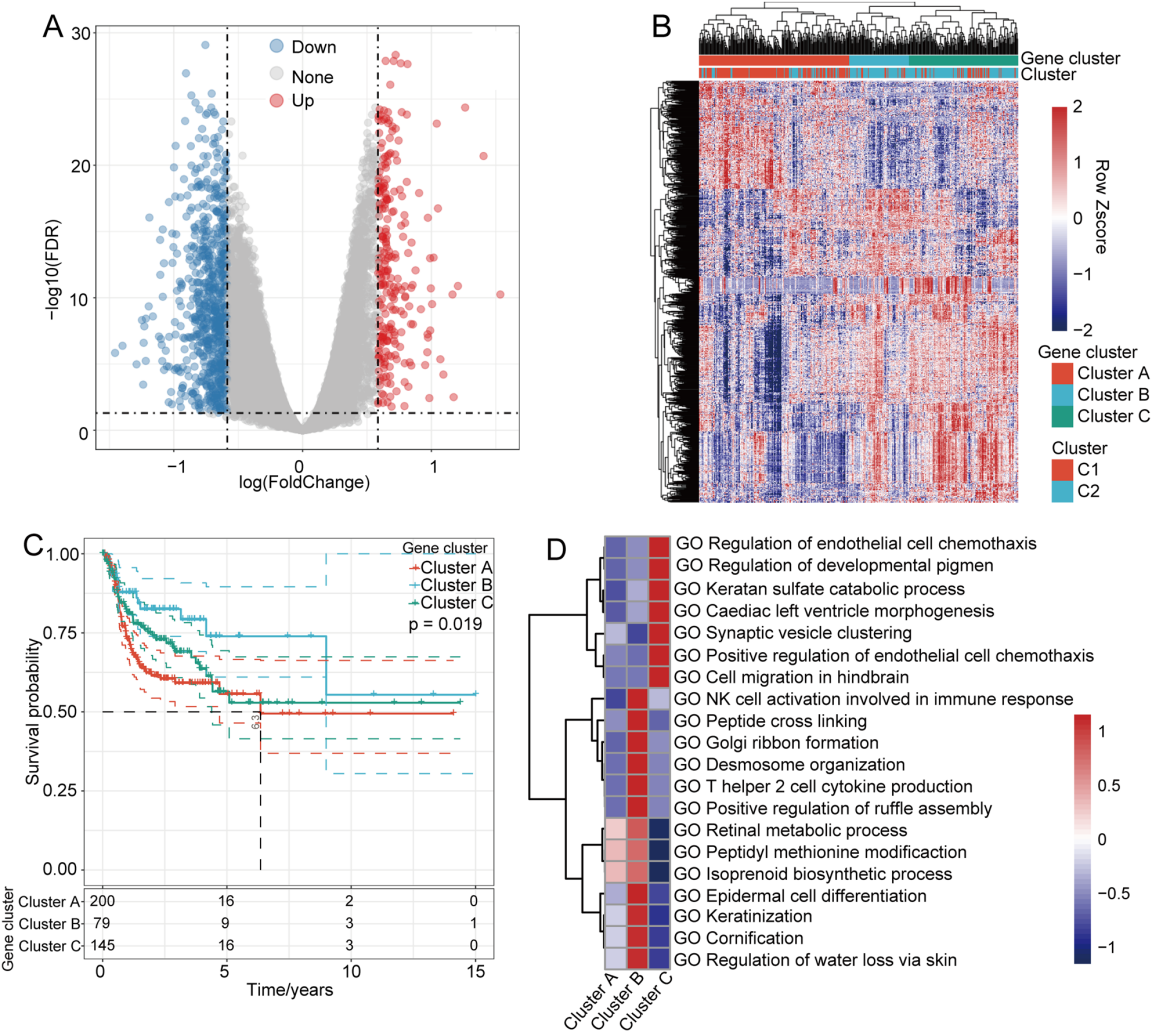
m6A-associated DEGs. (**A**) Volcano plot of m6A-associated DEGs. (**B**) Unsupervised cluster analysis diagram of m6A-associated DEGs. (**C**) KM curves of three m6A gene clusters. (**D**) A GSVA indicated that three clusters, A, B, and C, were enriched in different pathways.

In this analysis, we identified 945 DEGs between C1 and C2, which included 242 upregulated genes and 703 downregulated genes, as shown in the volcano plot (Fig. 4A). Furthermore, we performed unsupervised consensus clustering of the DEGs between the C1 and C2 subgroups and identified 3 clusters (cluster A, cluster B, and cluster C). C1 was mainly located in cluster A (Fig. 4B). According to the KM curve, cluster A presented the worst prognosis, whereas cluster C exhibited the best prognosis according to 15 years follow data (Fig. 4C). The adjusted pairwise comparisons demonstrated no significant differences between clusters C and B and between clusters A and C. Cluster B showed a shorter overall survival time than cluster A (p = 0.0066) (Supplementary Fig. 3D–F). In addition, a gene set variation analysis (GSVA) revealed that Clusters A, B, and C were enriched in significantly different pathways (Fig. 4D). Cluster A was mainly enriched in the retinal metabolic process, peptidyl methionine modification, and isoprenoid biosynthetic process categories. Cluster B was mainly enriched in natural killer cell activation involved in the immune response, peptide cross linking, Golgi ribbon formation, desmosome organization, T helper 2 cell cytokine production, positive regulation of ruffle assembly, epidermal cell differentiation, keratinization, cornification and regulation of water loss via skin. Cluster C was enriched in cell migration in the hindbrain, positive regulation of endothelial cell chemotaxis, synaptic vesicle clustering, cardiac lift ventricle morphogenesis, keratan sulfate catabolic process, regulation of developmental pigmentation and regulation of endothelial cell chemotaxis (Fig. 4D). To detect the m6A regulator expression levels in different clusters, we examined the distribution of these 20 genes in C1 and C2 (Supplementary Fig. 4A). We also examined the distribution of these 20 genes in clusters A, B, and C (Supplementary Fig. 4B).

### Construction and validation of an m6A regulator signature-based prognostic model

**Figure 5:**
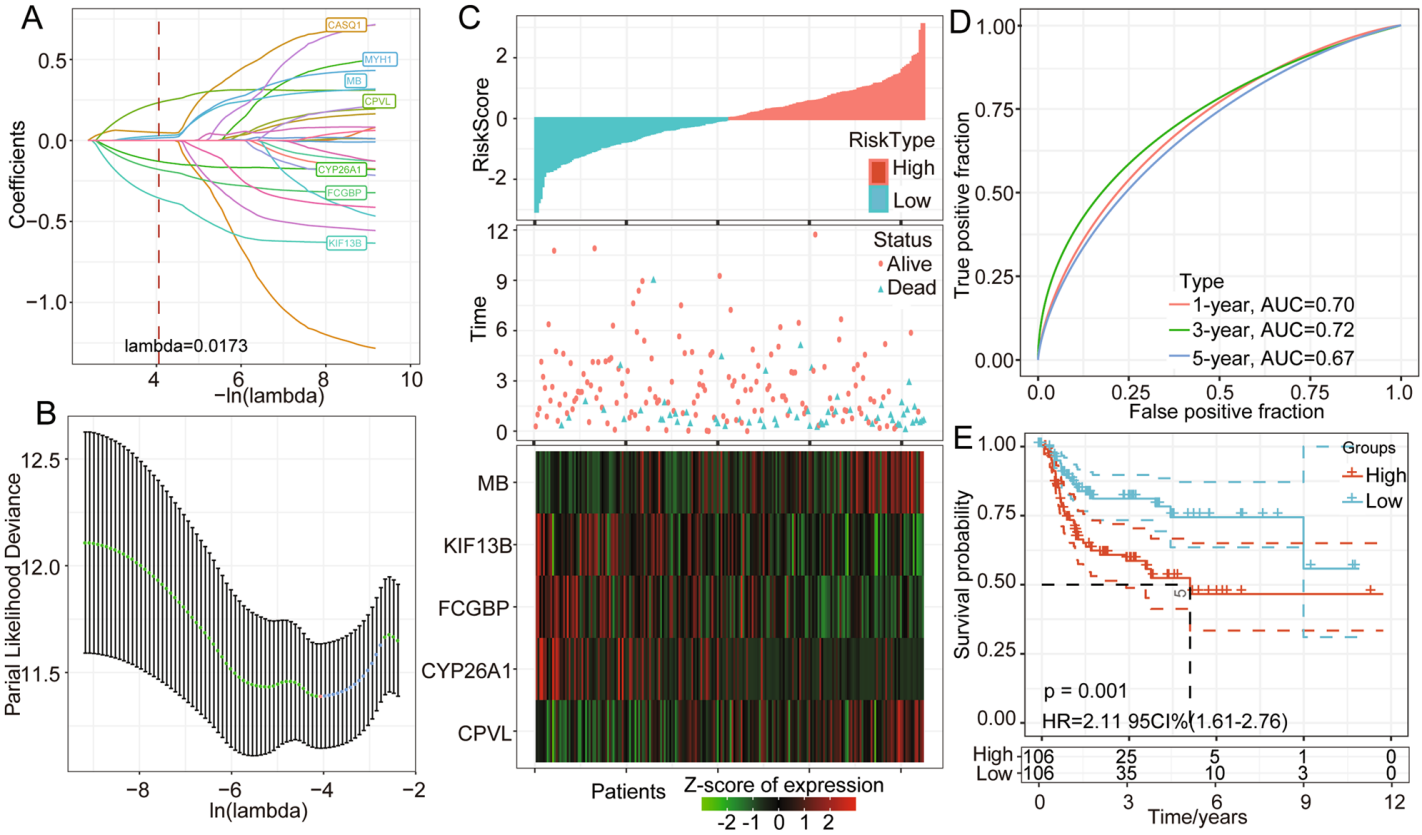
Construction and validation of a m6A phenotype-associated prognostic model. (**A**) The horizontal axis represents the log value of lambda, and the vertical axis represents the coefficient of the independent variable. (**B**) Confidence intervals for each lambda. (**C**) Risk score, survival time, survival status, and expression of 5 genes in the TCGA training group. (**D**) ROC curve and AUC of the 5-gene signature classification. (**E**) KM survival curve distribution between the high- and low-risk scores of the 5-gene signature in the training set.

**Figure 6:**
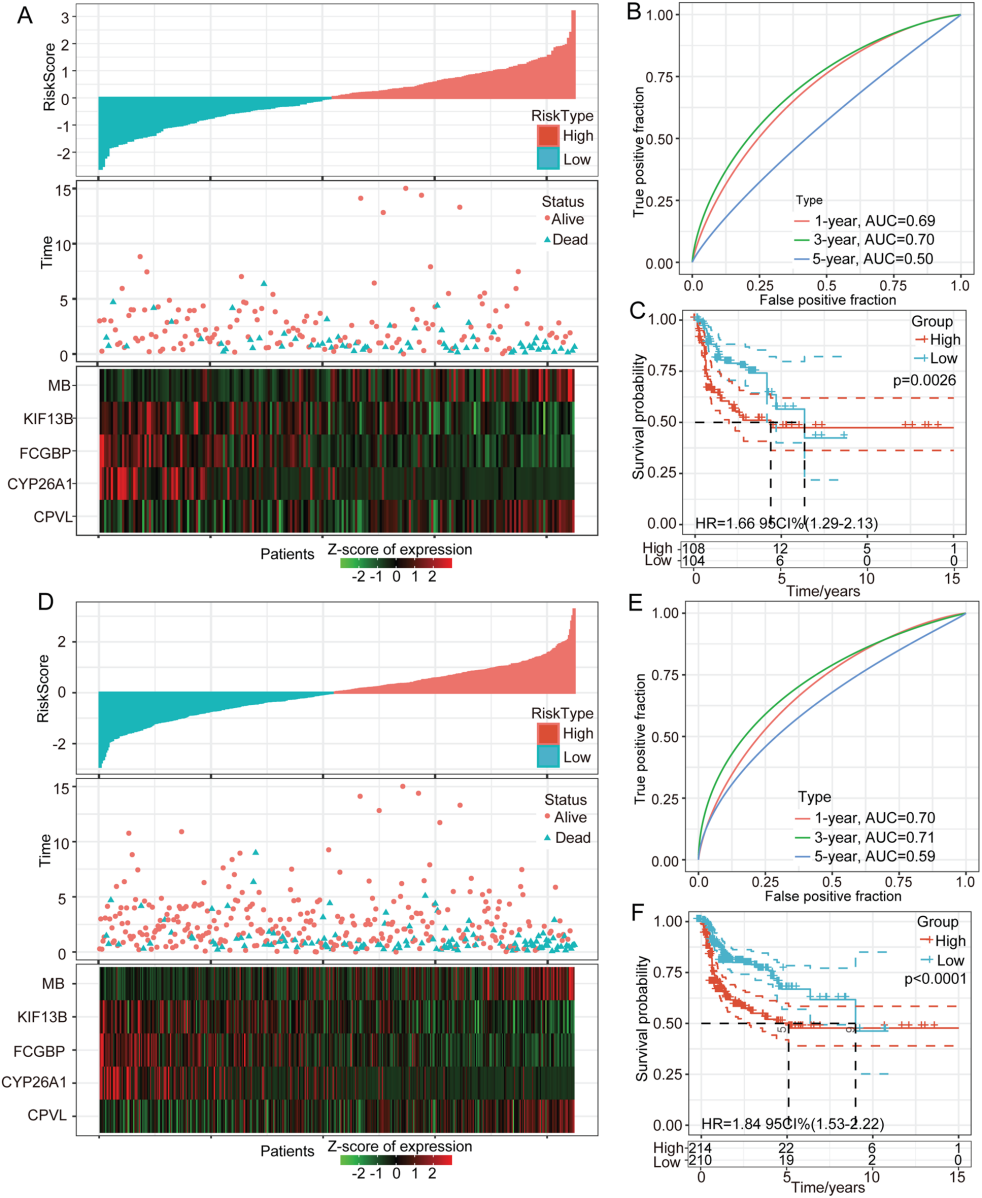
Validation of the m6A phenotype-associated prognostic model. (**A**) Risk score, survival time, survival status, five-gene expression, ACU curve, and KM curve of the TCGA validation dataset. (**B**) Risk score, survival time, survival status, five-gene expression, ACU curve, and KM curve of the TCGA all-sample validation dataset.

In detail, to better construct the prognostic model of m6A regulators, we applied Cox regression and lead absolute shrinkage and selection operator (LASSO) regression analysis and described the changing trajectory of each independent variable. The number of independent variable coefficients approaching zero increased with decrease in lambda (Fig. 5A). The confidence interval (CI) for each lambda was examined, as shown in Fig. 5B. The optimal model was acquired with a lambda value equal to 0.0173. We adopted the step AIC function from the MASS R package and identified 5 genes, namely, CYP26A1, KIF13B, CPVL, FCGBP, and MB. The 5-gene signature-based model was constructed according to the following equation: RiskScore = 0.178*CYP26A10.446*KIF13B + 0.305*CPVL0.238*FCGBP + 0.087*MB. The risk score landscape of each sample is illustrated in Fig. 5C. The AUC at 1, 3, and 5 years suggested that this 5-gene model presented good prognostic prediction efficacy (Fig. 5D). We calculated the risk score of each sample according to the expression level of the TCGA training dataset samples and obtained the risk scores of the samples. A Z score analysis of risk scores was then performed to categorize samples with scores > 0 into the high-risk group and those with scores < 0 into the low-risk group. The high-risk-score group presented a poorer prognosis than the low-risk-score group (uncorrected p = 0.001, HR = 2.11, 95% CI 1.61–2.76) (Fig. 5E). The TCGA gene profile was utilized as the validation dataset. The risk score distribution landscape and the AUC indicated a good and stable prediction efficiency (Fig. 6A,B). Consistent with the training set, the high-risk score subgroup showed a poor predicted prognosis (uncorrected p = 0.0026, HR = 1.66, 95% CI 1.29–2.13, Fig. 6C). To better evaluate the efficiency of the 5-gene model in the TCGA all-sample set, we calculated the risk score of each sample and the AUCs at 1, 3, and 5 years (Fig. 6D,E). The high-risk-score group presented a shorter survival time than the low-risk-score subgroup (p < 0.0001, HR = 1.84, 95% CI 1.53–2.22, Fig. 6F). The results agreed well with previous findings. Based on the previous 5-gene model, we also constructed an m6A regulator-associated gene model, and based on the expression of these m6A regulators, we classified HNSC patients into high- and low-risk score subgroups. The m6A score model efficiency was evaluated based on the TCGA training set and validated based on the TCGA validation set. A total of 424 samples from TCGA-HNSC were classified into a 212-sample training set and a 212-sample validation set. The detailed clinical information of the high- and low-m6A-score subgroups is illustrated in Supplementary Table 1. The high-m6A-score group presented a poorer prognosis than the low-m6A-score group (Supplementary Fig. 5A–D).

**Figure 7:**
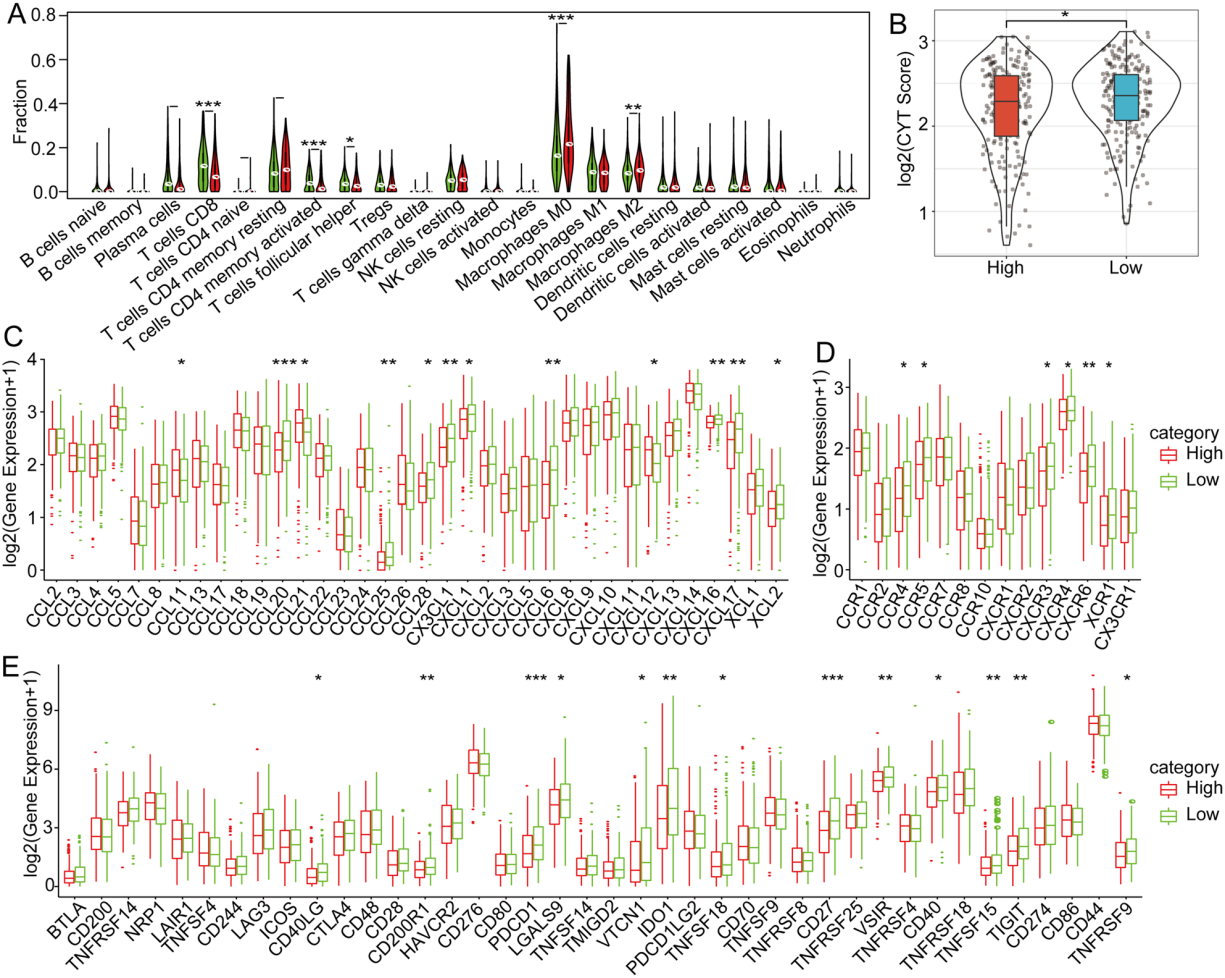
Correlation analysis of immune infiltration in the m6A-associated high- and low-risk-score groups. (**A**) Infiltration levels of 22 types of immune cells between the high- and low-m6A-risk score-based subgroups in the TCGA cohort. (**B**) CYT scores between the high- and low-m6A-risk-score subgroups. (**C**) Differences in chemokine expression between the high- and low-risk-score groups. (**D**) Distribution of chemokine receptor expression between the high- and low-risk-score groups. (**E**) Distribution of immune checkpoint gene expression between the high- and low-risk-score groups.

**Figure 8:**
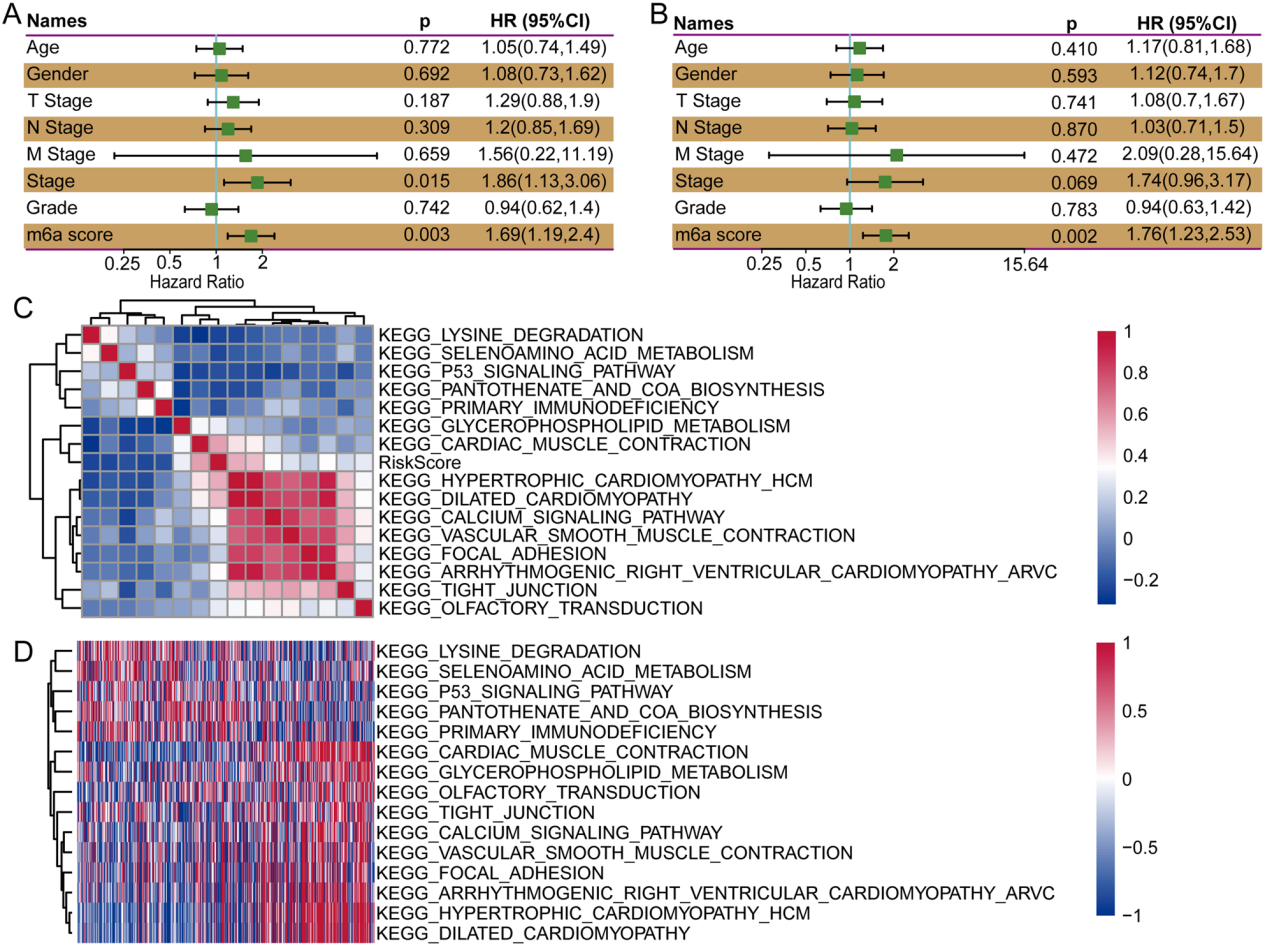
The m6A risk score model presents good independence in clinical application. (**A**) Univariate analysis of the m6A risk score model based on clinical factors. (**B**) Multivariate analysis of the m6A risk score model based on clinical factors. (**C**) Correlation coefficient clustering of risk scores and KEGG pathways. (**D**) Pathway enrichment analysis of different risk score subgroups.

The high-m6A-score group presented distinct immune-associated signatures. We calculated the infiltration levels of 22 immune cell types in the two m6A score-based groups, and the results demonstrated that 8 (36.36%) immune cell types showed a significant difference (Fig. 7A). We also performed a single-sample gene set enrichment analysis (ssGSEA) to calculate the IFNγ score of each patient, and significant differences in IFNγ scores were observed between the high- and low-score groups (Fig. 7B). To evaluate the differences in chemokine gene expression between the two groups, we calculated the chemokine gene expression levels and found that 12 chemokines showed significantly differential expression between the two groups (Fig. 7C). We also calculated the chemokine receptor gene expression levels and found that the expression levels of 6 of 18 (33.33%) genes were markedly different (Fig. 7D). Furthermore, we obtained 47 immune checkpoint genes and found that 14 (29.79%) immune checkpoint genes presented low expression levels in the high-m6A-score group (Fig. 7E). To investigate the correlation between m6A regulators and the hypoxia score, we performed GSEA and calculated the hypoxia score of each regulator. The results demonstrated that METTL3 (p < 0.05), ZC3H13 (p < 0.001), ALKBH5 (p < 0.001), FTO (p < 0.001), YTHDF1 (p < 0.001), YTHD3 (p < 0.001), HNRNPA2B1 (p < 0.01), FMR1 (p < 0.01), and ELAVL1 (p < 0.01) were significantly correlated with m6A regulators (Supplementary Fig. 6A) (the p value in this section is uncorrected). The m6A regulator-based cluster C had a markedly higher hypoxia score than cluster A (uncorrected p = 0.001). The high-m6A-score subgroup exhibited a higher hypoxia score than the low-m6A-score subgroup. The hypoxia score did not significantly differ between C1 and C2 (Supplementary Fig. 6B). We performed univariate and multivariate Cox regression analyses to evaluate the independence of the m6A score signature model in terms of clinical application. The univariate Cox regression analysis showed that the m6A score was significantly correlated with survival, and the corresponding multivariate Cox regression analysis revealed that the m6A score-based group (uncorrected p < 0.05, HR = 1.76, 95% CI 1.23–2.53) was significantly correlated with survival (Fig. 8A,B). These outcomes revealed that the m6A score model can exhibit good prognostic prediction value in the clinic.

### Biological functional enrichment in the m6A score model

To explore the relationship between the m6A score and associated biological functions, we performed ssGSEA and determined that five functional pathways were negatively correlated with the m6A score and that 10 pathways were positively correlated with the sample m6A score. In addition, a Kyoto Encyclopedia of Genes and Genomes (KEGG) analysis was performed in this study to investigate the enriched signaling pathways^10^. The results from the KEGG enrichment-based cluster analysis of the pathways are illustrated in Fig. 8C. The KEGG pathways cardiac muscle construction, glycerophospholipid metabolism and olfactory transduction showed increased enrichment in the high-m6A-score group (Fig. 8D).

## Discussion

Epigenetic modifications have emerged as abundant modifications throughout the transcriptome and play very important roles in genome evolution and innovation^11^. m6A plays a predominant role in controlling gene expression and influencing physiological and pathological processes^12^. Increasing evidence demonstrates that alterations in m6A regulators are novel and decisive factors in tumor progression, chemotherapy resistance, and immunotherapy responses^13^. m6A modification promotes mitochondrial energy metabolism in the pathogenesis of colorectal cancer by both stabilizing the ZFAS1/OLA1 axis and activating the Warburg effect^14^. KIAA1429 functions as a potential prognostic marker in colorectal cancer because it activates proliferation by downregulating WEE1 expression in an m6A-independent manner^6^. Zhang et al. revealed that m6A regulators serve as therapeutic targets for overcoming chemotherapy resistance in small-cell lung cancer patients^15^. The N6-methyladenosine reader IMP2 stabilizes the ZFAS1/OLA1 axis and activates the Warburg effect, which is implicated in colorectal cancer. In our analysis, we comprehensively investigated m6A regulator alterations and their prognostic roles and constructed a risk score model based on HNSCC patient-based public databases. To better understand the crucial functions of m6A regulators in HNSCC progression and proliferation, we classified m6A regulators into two clusters. Cluster 1 exhibited a poor prognosis and was characterized by decreases in the HIPPO-, MYC-, NOTCH-, NRF1-, TGF-beta-, PIK3-, and RAS-associated tumor pathways. We also identified three clusters of m6A phenotype-associated DEGs. These three clusters presented significantly different prognoses and enrichment pathways. Furthermore, we constructed five hub gene-based risk score models, and validation of these models revealed their good clinical stability. Furthermore, we built and validated an m6A score model with good stability. RNA m6A modification is abundant in eukaryotes, bacteria and archaea^16,17^. This RNA modification mainly functions in mRNAs and plays a very important role in the metabolism and function of mRNAs. Recent studies have revealed that m6A regulators are involved in the occurrence and development of cancers^18^. These regulators mainly have three types of functions: “writers”, “erasers” and “readers”. m6A regulators are dynamic and reversible epigenetic modifications that are able to regulate the ability of cells to differentiate and regenerate^18^. The m6A-related DEGs might be involved in abundant functions^19^. Therefore, exploring the m6A modification mechanism in cancer initiation and progression is of great importance and might pave the way for further HNSCC diagnosis and treatment.

Convincing evidence indicates that m6A regulators are involved in a variety of types of pathological and physiological immune cell infiltration and immune responses and safeguard homeostasis and tumor immunosurveillance functions^8^. Zhou et al. revealed that the m6A regulator ALKBH5 plays an unexpectedly specific role in controlling the pathogenicity of CD4 T cells during autoimmunity^20^. Our study showed that a high m6A risk score indicates poor clinical characteristics, immune infiltration levels, and chemokine, chemokine receptor, and immune checkpoint gene expression. Lin et al. reported that the crosstalk of RNA modifications is also involved in immune infiltration characteristics^21^. Ni et al. demonstrated that in bladder cancer, METTL3 resists the cytotoxicity of CD8+ T cells by regulating PD-L1 expression^22^. Gao et al. found that m6A methylation also plays a very important role in the regulation of tumor malignancy and antitumor immunomodulation^23^. Yi et al. demonstrated that m6A regulators exert a very important effect on immune cell infiltration in HNSCC^24^. Jin et al. reported that the m6A regulator ALKBH5 might suppress tumor progression in the immune microenvironment through the RIG-1/IFNA axis^25^.

Yang et al. revealed that m6A regulators are closely related to the tumor microenvironment and might affect immunotherapy and chemical strategies for HNSCC^26^. These findings pave the way for novel HNSCC diagnosis and treatment methods. In addition, further exploration of HNSCC might identify more therapeutic targets for early diagnosis and treatment. Despite this finding, larger samples and independent validations are still needed to develop prognostic biomarkers of the immune features of HNSCC in future studies. Thus, the crucial regulatory mechanism of m6A modification in HNSCC progression and the immune microenvironment is quite unclear in terms of biological and pathological processes, and further research in both laboratory and clinical settings is needed.

In summary, this model was found to exhibit good abilities for HNSCC prognosis prediction, immune infiltration level evaluation and risk factor assessment.

## Methods and materials

### Databases

Fragments per kilobase million (FPKM) data for HNSCC were obtained from the TCGA HNSCC cohort (https://portal.gdc.cancer.gov/projects/TCGA-HNSC). Count data were converted to transcripts per million (TPM) values, and all subsequent analyses were performed using the TPM values. Some samples with no corresponding mutation data (or gene expression profile data) were removed. Samples without a living status and an alive time less than 0 were eliminated. In this analysis, a total of 424 tumor samples and 44 normal samples with gene expression profiles and corresponding adjacent normal tissues were included. In total, 508 HNSCC patients with mutations in TCGA databases were selected for further analysis.

### Identification of alterations of m6A regulators

We downloaded HNSCC single nucleotide variation (SNV) and copy number variation (CNV) profiles from TCGA. According to the degree of CNV changes, we classified CNVs into three types: amplifications (segment mean > 0.2), duplications (− 0.2 < segment mean < 0.2), and deletions (segment mean < − 0.2).

### Cox regression analysis

Variables showing notable differences in a univariate Cox regression analysis were applied in this analysis. Variables with a p value < 0.05 in the univariate Cox analysis were subjected to stepwise multivariate Cox regression analysis.

### ConsensusClusterPlus

ConsensusClusterPlus is a useful technique in cancer research in which intrinsic groups share biological characteristics, with confidence assessments and item tracking^27^. Cluster analysis was performed using the ConsensusClusterPlus package in R (https://www.r-project.org/)^27^. The following key parameters were applied to evaluate the optimal number of clusters (k): clusterAlg = "pam", distance = "spearman", cumulative distribution function (CDF) and relative expression level according to the area under the curve (AUC).

### CIBERSORT

Tumor-infiltrating immune cells (TIICs) in HNSCC cohorts were recognized by Cell type Identification by Estimating Relative Subsets of RNA Transcripts (CIBERSORT). We adopted the ‘CIBERSORT’ R package (CIBERSORT R script v1.03; http://cibersort.stanford.edu/) to examine the relative expression of immune-associated genes in each sample.

### GSVA

The gene sets used in the GSVA were downloaded from the GSEA molecular database. The GSVA was conducted using the GSVA package in R based on log2 (normalized counts + 1) expression values. We identified p < 0.05 as the threshold for the replication of gene sets. First, 424 samples from the TCGA-HNSC dataset were divided into training and validation sets. To calculate each coefficient, we applied the summaryBy function (in the doBy package) with the frac parameter set to 0.5^28^. In total, we selected 212 samples in the training set and 212 samples in the validation set, as shown in Table 2. The sample information of the training and verification sets of the TCGA data that were finally obtained is shown in Table 2. The chi-square test was used to test the samples belonging to the training and validation sets, and the results showed that there was no bias in our grouping and no significant difference between groups (p > 0.05). We applied the univariate Cox proportional risk regression model to 853 DEGs. In the analysis of the training set using the survival R package function coxph, p < 0.05 was selected as the threshold.

**Table 2:** The TCGA training set and TCGA test set.

Clinical features	TCGA-training set	TCGA-validation set	p value
**OS**			
0	152	144	0.459
1	60	68	
**T stage**			
T1	14	14	0.9741
T2	60	57	
T3	56	60	
T4	82	81	
**Stage**			
I	13	8	0.6265
II	32	34	
III	36	42	
IV	131	128	
**Grade**			
G1	26	25	0.4937
G2	135	132	
G3	49	55	
G4	2	0	
**Gender**			
Female	58	56	0.9128
Male	154	156	
**Age**			
≤ 60	99	103	0.7705
> 60	113	109	

### LASSO regression analysis

The m6A regulator-based signatures might have important prognostic value in HNSCC. First, two molecular subtypes were constructed based on the consensus clustering of m6A-related genes, and the differentially expressed genes related to the m6A subtype were screened by differential analysis. We applied LASSO regression analysis to build prediction models based on the training dataset and validated their predictive effectiveness using the validation cohort. The genes associated with prognosis were screened by univariate Cox analysis of the initial input of 27 genes, namely, CYP26A1, KIF13B, CPVL, CMYA5, FCGBP, XIRP2, NEB, CASQ1, MYH2, NRAP, MYH7, LMOD2, ACTN2, MYOT, HSPB7, APOBEC2, MYL1, MYH1, ACTA1, MB, MYOZ1, CSRP3, MYL2, COX6A2, SMPX, CKM and SLN. Then, LASSO analysis and multivariate Cox analysis were applied to construct a risk-score based prognoistic model. Among these differentially expressed genes, five key genes were screened by univariate Cox analysis.

The glmnet R package was used for LASSO Cox variable selection and model building. The survival R package was used for the comparison of survival curves. Survival analysis based on the Kaplan–Meier (KM) curve was applied to evaluate prognosis.

### Statistics

Statistical analyses and data plotting were performed using the R program (3.6.2)^29^. Fisher’s exact and equal-variance t tests were used in the group comparisons of categorical and continuous variables, respectively. Spearman’s correlation analysis test was applied to evaluate the correlation relationships of different cancer types. p ≤ 0.05 indicates statistical significance.

## Supplementary Information

Supplementary Information 1.

Supplementary Information 2.

## Data Availability

The datasets used and/or analyzed during the current study are available from the corresponding author upon request.

## References

[1] Dierks D (2021). Multiplexed profiling facilitates robust m6A quantification at site, gene and sample resolution. Nat. Methods.

[2] Gu C (2019). Mettl14 inhibits bladder TIC self-renewal and bladder tumorigenesis through N-methyladenosine of Notch1. Mol. Cancer.

[3] Yin H (2021). M6A RNA methylation-mediated RMRP stability renders proliferation and progression of non-small cell lung cancer through regulating TGFBR1/SMAD2/SMAD3 pathway. Cell Death Differ..

[4] Liu H-T (2021). lncRNA THAP7-AS1, transcriptionally activated by SP1 and post-transcriptionally stabilized by METTL3-mediated m6A modification, exerts oncogenic properties by improving CUL4B entry into the nucleus. Cell Death Differ..

[5] Guo Y (2021). Comprehensive analysis of m6A RNA methylation regulators and the immune microenvironment to aid immunotherapy in pancreatic cancer. Front. Immunol..

[6] Ma L (2021). KIAA1429 is a potential prognostic marker in colorectal cancer by promoting the proliferation via downregulating WEE1 expression in an m6A-independent manner. Oncogene.

[7] Liu T (2021). METTL14 suppresses growth and metastasis of renal cell carcinoma by decreasing long non-coding RNA NEAT1. Cancer Sci..

[8] Song H (2021). METTL3-mediated mA RNA methylation promotes the anti-tumour immunity of natural killer cells. Nat. Commun..

[9] Zhang Z (2021). mA regulator expression profile predicts the prognosis, benefit of adjuvant chemotherapy, and response to anti-PD-1 immunotherapy in patients with small-cell lung cancer. BMC Med..

[10] Kanehisa M, Furumichi M, Sato Y, Ishiguro-Watanabe M, Tanabe M (2021). KEGG: Integrating viruses and cellular organisms. Nucleic Acids Res..

[11] Miao Z, Zhang T, Xie B, Qi Y, Ma C (2021). Evolutionary implications of the RNA N6-methyladenosine methylome in plants. Mol. Biol. Evol..

[12] Huang W (2021). N6-methyladenosine methyltransferases: Functions, regulation, and clinical potential. J. Hematol. Oncol..

[13] Huang H, Weng H, Chen J (2020). mA modification in coding and non-coding RNAs: Roles and therapeutic implications in cancer. Cancer Cell.

[14] Lu S (2021). N6-methyladenosine reader IMP2 stabilizes the ZFAS1/OLA1 axis and activates the Warburg effect: Implication in colorectal cancer. J. Hematol. Oncol..

[15] Zhang Z (2021). mA regulators as predictive biomarkers for chemotherapy benefit and potential therapeutic targets for overcoming chemotherapy resistance in small-cell lung cancer. J. Hematol. Oncol..

[16] Zhou H, Mao L, Xu H, Wang S, Tian J (2022). The functional roles of m(6)A modification in T lymphocyte responses and autoimmune diseases. Cytokine Growth Factor Rev..

[17] Alarcón CR, Lee H, Goodarzi H, Halberg N, Tavazoie SF (2015). N6-methyladenosine marks primary microRNAs for processing. Nature.

[18] Xue C (2022). Role of main RNA modifications in cancer: N(6)-methyladenosine, 5-methylcytosine, and pseudouridine. Signal Transduct. Target Ther..

[19] Nombela P, Miguel-López B, Blanco S (2021). The role of m(6)A, m(5)C and Ψ RNA modifications in cancer: Novel therapeutic opportunities. Mol. Cancer.

[20] Zhou J (2021). mA demethylase ALKBH5 controls CD4 T cell pathogenicity and promotes autoimmunity. Sci. Adv..

[21] Lin Q, Ni H, Zheng Z, Zhong J, Nie H (2022). Cross-talk of four types of RNA modification writers defines the immune microenvironment in severe asthma. Ann. N Y Acad. Sci..

[22] Ni Z (2022). JNK signaling promotes bladder cancer immune escape by regulating METTL3-mediated m6A modification of PD-L1 mRNA. Cancer Res..

[23] Gao Y (2022). Single-cell N(6)-methyladenosine regulator patterns guide intercellular communication of tumor microenvironment that contribute to colorectal cancer progression and immunotherapy. J. Transl. Med..

[24] Yi L, Wu G, Guo L, Zou X, Huang P (2020). Comprehensive analysis of the PD-L1 and immune infiltrates of m(6)A RNA methylation regulators in head and neck squamous cell carcinoma. Mol. Ther. Nucleic Acids.

[25] Jin S (2022). The m6A demethylase ALKBH5 promotes tumor progression by inhibiting RIG-I expression and interferon alpha production through the IKKε/TBK1/IRF3 pathway in head and neck squamous cell carcinoma. Mol. Cancer.

[26] Yang Z (2021). Comprehensive analysis of m(6)A regulators characterized by the immune cell infiltration in head and neck squamous cell carcinoma to aid immunotherapy and chemotherapy. Front. Oncol..

[27] Wilkerson MD, Hayes DN (2010). ConsensusClusterPlus: A class discovery tool with confidence assessments and item tracking. Bioinformatics (Oxford, England).

[28] Chavan SG, Duursma RA, Tausz M, Ghannoum O (2019). Elevated CO2 alleviates the negative impact of heat stress on wheat physiology but not on grain yield. J. Exp. Bot..

[29] Zhu J, Xiao J, Wang M, Hu D (2020). Pan-cancer molecular characterization of Ma regulators and immunogenomic perspective on the tumor microenvironment. Front. Oncol..

