# Supplementary material for: m6A RNA methylation regulator-related signatures exhibit good prognosis prediction ability for head and neck squamous cell carcinoma
Source: Sci Rep. 2022 Sep 29;12:16358. doi: 10.1038/s41598-022-20873-6 (PMC9523032; doi:10.1038/s41598-022-20873-6)
Supplement: Supplementary file 2 — Supplementary Information 2. [file 41598_2022_20873_MOESM2_ESM.docx]

**Supplementary Figure 1**

**
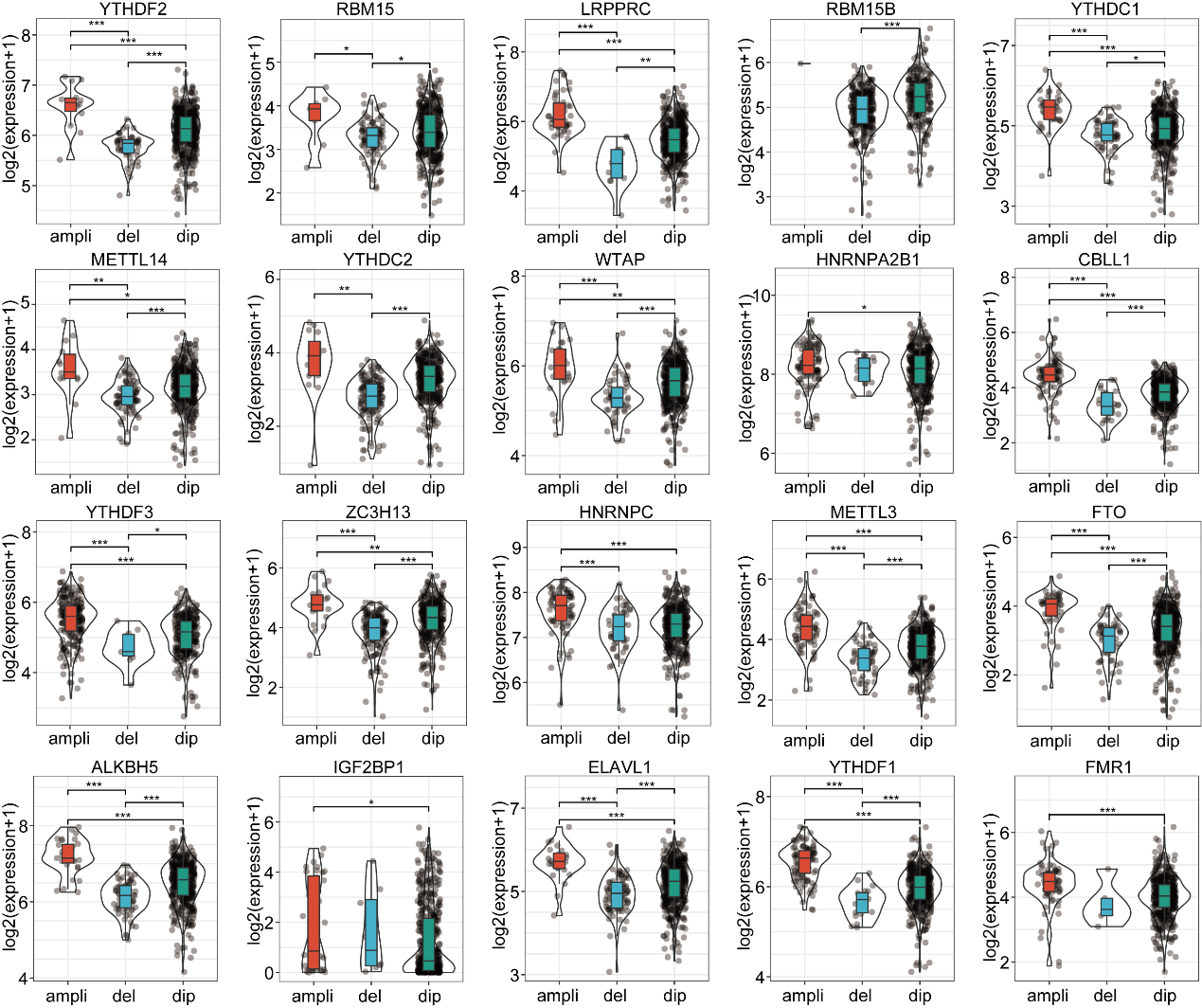
**

**Supplementary Figure 2**

**
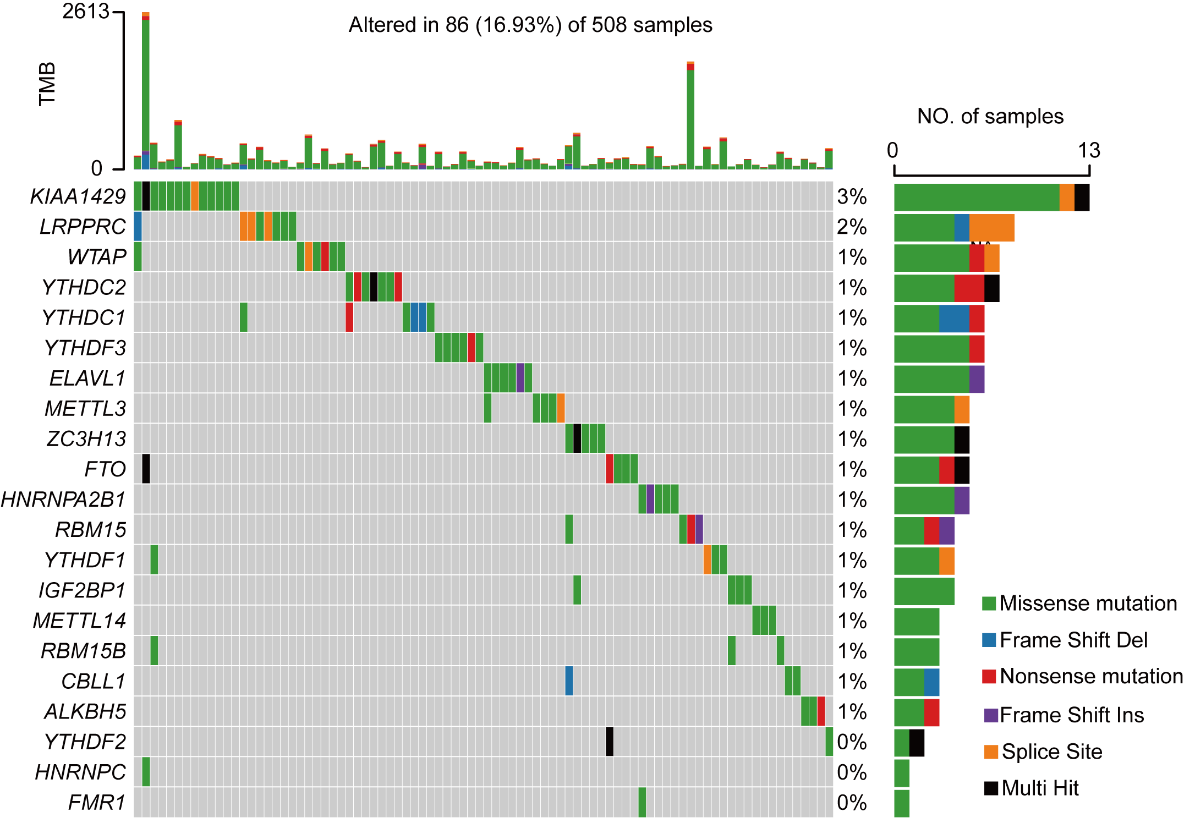
**

**Supplementary Figure 3**

**
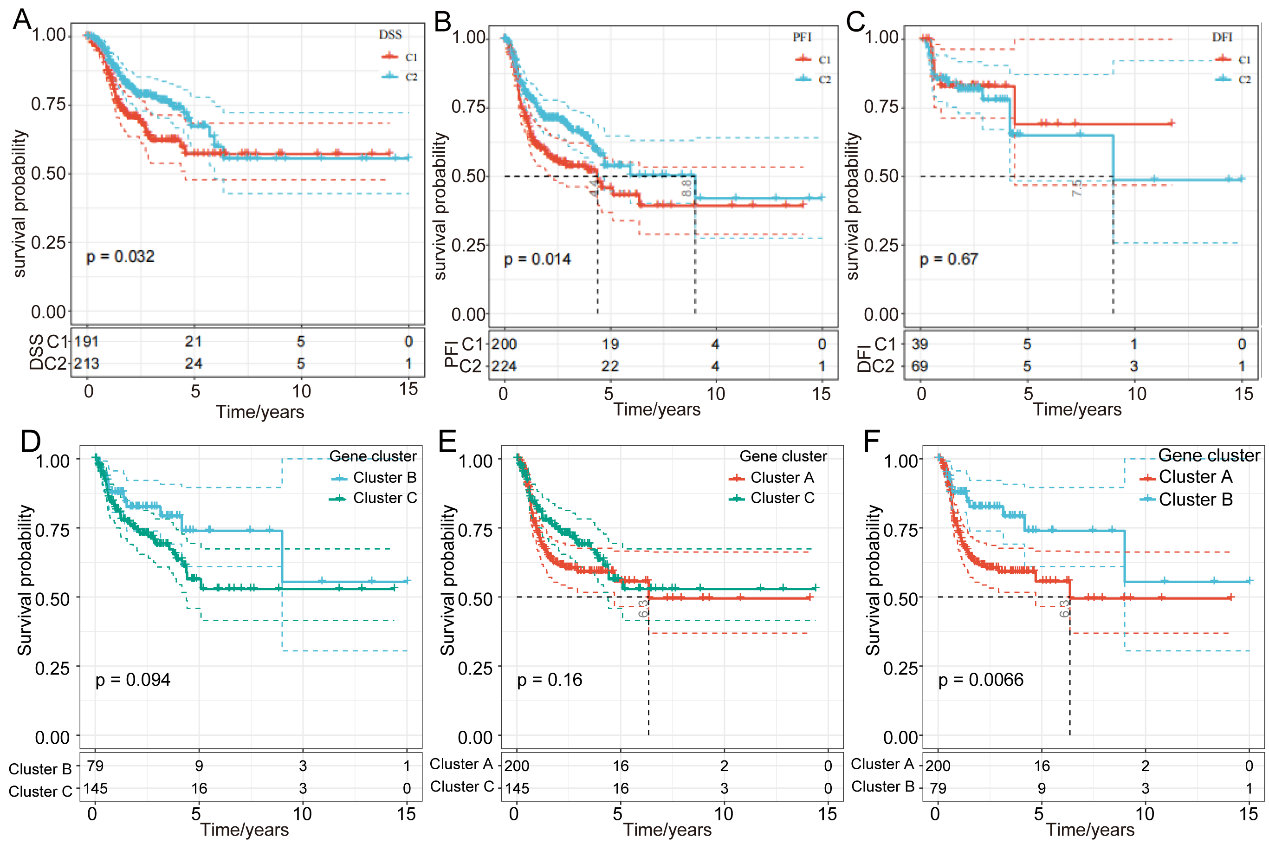
**

**Supplementary Figure 4**

**
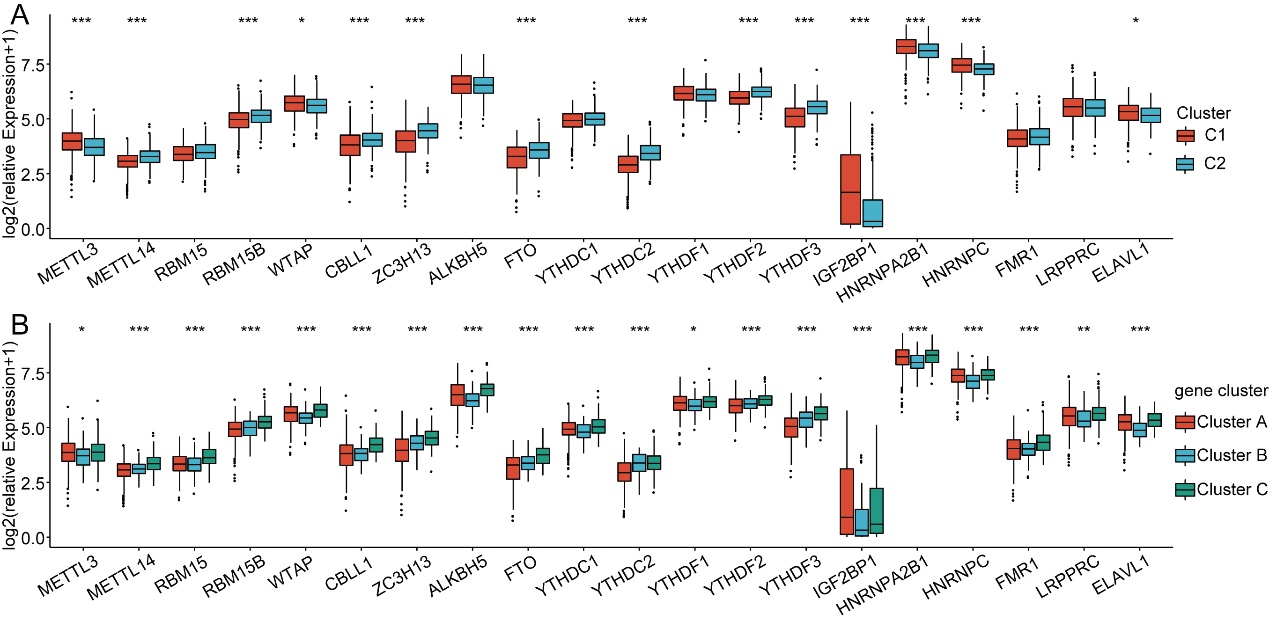
**

**Supplementary Figure 5**

**
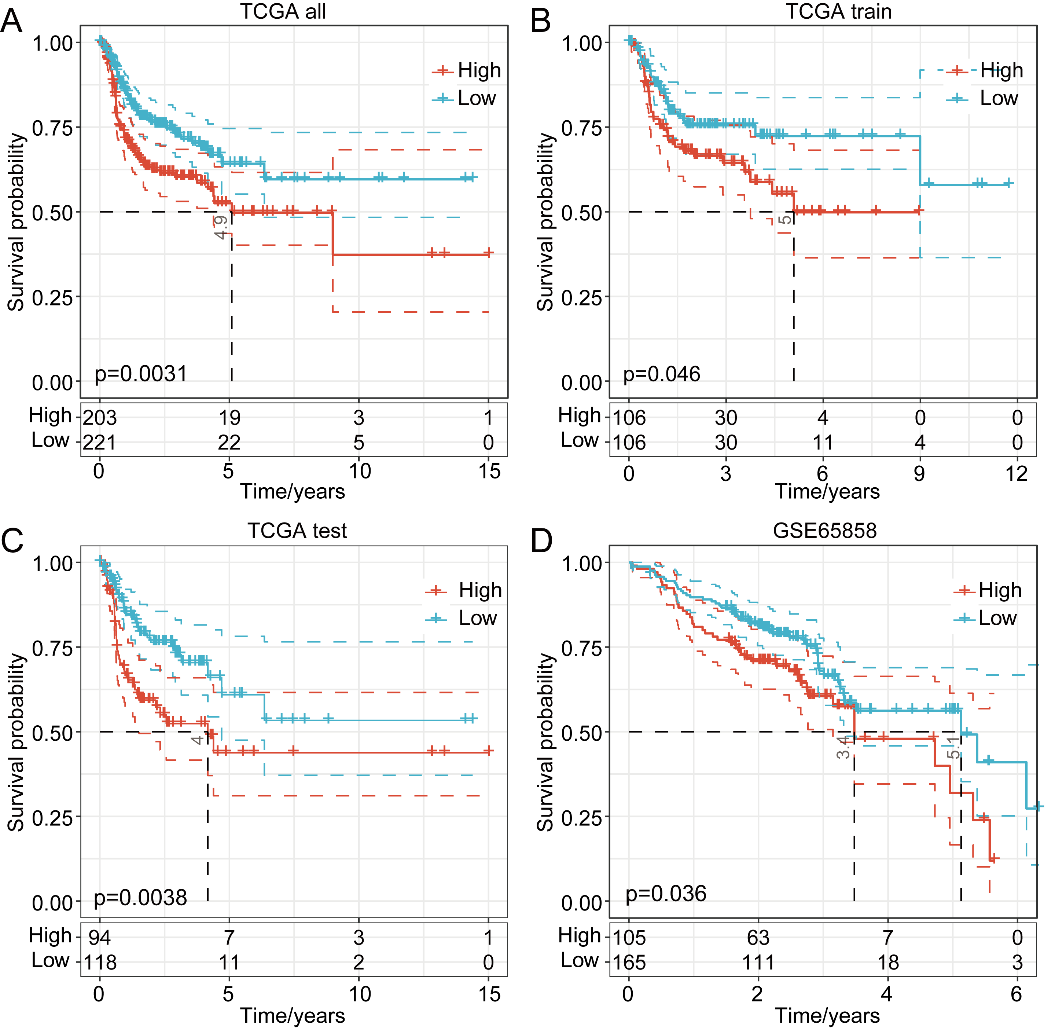
**

**Supplementary Figure 6**

**
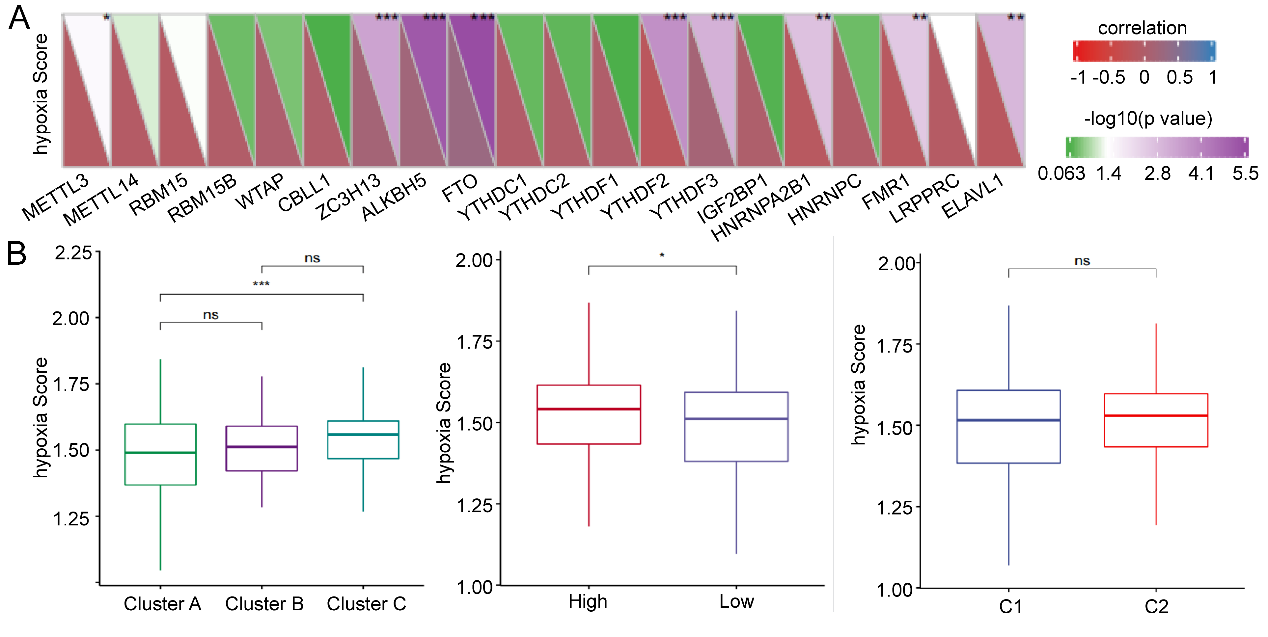
**

**Supplementary Figure legends**

**Supplementary Figure 1** The relationship between CNV mutations and m6A phenotype-associated genes.

**Supplementary Figure 2** The relationship between SNV mutations and m6A phenotype-associated genes.

**Supplementary Figure 3** Clinical endpoint evaluation between C1 and C2. **A.** C2 cluster has significantly improved the DSS compared with C1. **B.** C2 cluster has longer PFI compared with C1. **C.** The DFI between C1 and C2 showed no significant difference between C1 and C2 cluster. **D-F.** The adjusted pairwise comparisons demonstrated no significant differences between clusters C and B and between clusters A and C. Cluster B showed a shorter overall survival time than cluster A.

**Supplementary Figure 4** The distribution of m6A regulator gene expression in m6A clusters (C1 and C2) (A) and m6A-related gene clusters (Cluster A, Cluster B, Cluster C) (B).

**Supplementary Figure 5** There was a significant difference between the high and low groups in the training set and validation sets (TCGA all, TCGA train, TCGA test, and GSE65858). (A-D) There was a significant difference between the high- and low-score groups, and the high-score group presented a poor prognosis.

**Supplementary Figure 6** The correlation m6A regulators and hypoxia score. A. The METTL3, ZC3H13, ALKBH5, FTO, YTHDF1, YTHD3, HNRNPA2B1, FMR1, and ELAVL1 were significantly correlated with m6A regulators. B. The m6Aregulators based cluster C had remarkable higher hypoxia score compared with cluster A. The high m6A score subgroup obtained higher hypoxia score compared with low m6A score. The hypoxia score between C1 and C2 had no significant difference.

**Supplementary Table 1. The detailed clinical information of between C1 and C2**

|  | **Cluster** | |  |  |  |
| --- | --- | --- | --- | --- | --- |
| **TCGA** | **C1 (n=200)** | **C2 (n=224)** | **Total (n=424)** | **p value** | **FDR** |
| Radiation therapy | | | | 9.00E-12 | 8.10E-11 |
| NO | 28(6.60%) | 25(5.90%) | 53(12.50%) |  |  |
| YES | 76(17.92%) | 23(5.42%) | 99(23.35%) |  |  |
| HPV status |  |  |  | 0.88 | 1 |
| Negative | 30(7.08%) | 35(8.25%) | 65(15.33%) |  |  |
| Positive | 10(2.36%) | 9(2.12%) | 19(4.48%) |  |  |
| T stage | | | | 4.00E-03 | 0.03 |
| T1 | 9(2.12%) | 19(4.48%) | 28(6.60%) |  |  |
| T2 | 43(10.14%) | 74(17.45%) | 117(27.59%) |  |  |
| T3 | 56(13.21%) | 60(14.15%) | 116(27.36%) |  |  |
| T4 | 92(21.70%) | 71(16.75%) | 163(38.44%) |  |  |
| N stage | | | | 0.56 | 1 |
| N0 | 95(22.41%) | 122(28.77%) | 217(51.18%) |  |  |
| N1 | 38(8.96%) | 37(8.73%) | 75(17.69%) |  |  |
| N2 | 64(15.09%) | 62(14.62%) | 126(29.72%) |  |  |
| N3 | 3(0.71%) | 3(0.71%) | 6(1.42%) |  |  |
| M stage | | | | 0.54 | 1 |
| M0 | 197(46.46%) | 223(52.59%) | 420(99.06%) |  |  |
| M1 | 3(0.71%) | 1(0.24%) | 4(0.94%) |  |  |
| Stage | | | | 7.00E-03 | 0.05 |
| I | 6(1.42%) | 15(3.54%) | 21(4.95%) |  |  |
| II | 21(4.95%) | 45(10.61%) | 66(15.57%) |  |  |
| III | 37(8.73%) | 41(9.67%) | 78(18.40%) |  |  |
| IV | 136(32.08%) | 123(29.01%) | 259(61.08%) |  |  |
| Grade | | | | 0.02 | 0.09 |
| G1 | 14(3.30%) | 37(8.73%) | 51(12.03%) |  |  |
| G2 | 128(30.19%) | 139(32.78%) | 267(62.97%) |  |  |
| G3 | 57(13.44%) | 47(11.08%) | 104(24.53%) |  |  |
| G4 | 1(0.24%) | 1(0.24%) | 2(0.47%) |  |  |
| Gender | | | | 0.04 | 0.21 |
| Female | 44(10.38%) | 70(16.51%) | 114(26.89%) |  |  |
| Male | 156(36.79%) | 154(36.32%) | 310(73.11%) |  |  |
| Age | | | | 0.59 | 1 |
| <=60 | 92(21.70%) | 110(25.94%) | 202(47.64%) |  |  |
| >60 | 108(25.47%) | 114(26.89%) | 222(52.36%) |  |  |

**Supplementary Table 2 The detailed clinical information of between high and low m6A scores subgroups**

| **GSE65858** | **Low (n=165)** | **High (n=105)** | **Total (n=270)** | **p value** | **FDR** |
| --- | --- | --- | --- | --- | --- |
| Gender |  |  |  | 0.56 | 1 |
| Female | 31(11.48%) | 16(5.93%) | 47(17.41%) |  |  |
| Male | 134(49.63%) | 89(32.96%) | 223(82.59%) |  |  |
| Smoking |  |  |  | 0.7 | 1 |
| No | 31(11.48%) | 17(6.30%) | 48(17.78%) |  |  |
| Yes | 134(49.63%) | 88(32.59%) | 222(82.22%) |  |  |
| HPV |  |  |  | 0.24 | 1 |
| HPV16 | 40(14.81%) | 20(7.41%) | 60(22.22%) |  |  |
| Negative | 115(42.59%) | 81(30.00%) | 196(72.59%) |  |  |
| Other HPV | 10(3.70%) | 3(1.11%) | 13(4.81%) |  |  |
| T Stage |  |  |  | 0.38 | 1 |
| T1 | 20(7.41%) | 15(5.56%) | 35(12.96%) |  |  |
| T2 | 54(20.00%) | 26(9.63%) | 80(29.63%) |  |  |
| T3 | 31(11.48%) | 27(10.00%) | 58(21.48%) |  |  |
| T4 | 60(22.22%) | 37(13.70%) | 97(35.93%) |  |  |
| N Satge |  |  |  | 0.99 | 1 |
| N0 | 57(21.11%) | 37(13.70%) | 94(34.81%) |  |  |
| N1 | 19(7.04%) | 13(4.81%) | 32(11.85%) |  |  |
| N2 | 82(30.37%) | 50(18.52%) | 132(48.89%) |  |  |
| N3 | 7(2.59%) | 5(1.85%) | 12(4.44%) |  |  |
| Stage |  |  |  | 0.86 | 1 |
| I | 12(4.44%) | 6(2.22%) | 18(6.67%) |  |  |
| II | 21(7.78%) | 16(5.93%) | 37(13.70%) |  |  |
| III | 24(8.89%) | 13(4.81%) | 37(13.70%) |  |  |
| IV | 108(40.00%) | 70(25.93%) | 178(65.93%) |  |  |
